# Sensory modality defines the relation between EEG Lempel–Ziv diversity and meaningfulness of a stimulus

**DOI:** 10.1038/s41598-023-30639-3

**Published:** 2023-03-01

**Authors:** Paweł Orłowski, Michał Bola

**Affiliations:** Laboratory of Brain Imaging, Nencki Institute of Experimental Biology of Polish Academy of Sciences, 3 Pasteur Street, 02-093 Warsaw, Poland

**Keywords:** Cognitive neuroscience, Sensory processing, Visual system

## Abstract

Diversity of brain activity is a robust neural correlate of global states of consciousness. It has been proposed that diversity measures specifically reflect the temporal variability of conscious experience. Previous studies supported this hypothesis by showing that perception of meaningful visual stimuli causes richer, more-variable experiences than perception of meaningless stimuli, and this is reflected in greater brain signal diversity. To investigate whether this relation is consistent across sensory modalities, to participants we presented three versions of naturalistic visual and auditory stimuli (videos and audiobooks) that varied in the amount of meaning (original, scrambled, and noise), while recording electroencephalographic signals. We report three main findings. First, greater meaningfulness of visual stimuli was related to higher Lempel–Ziv diversity of EEG signals, but the opposite effect was found in the auditory modality. Second, visual perception was related to generally higher EEG diversity than auditory perception. Third, perception of meaningful visual stimuli and auditory stimuli respectively resulted in higher and lower EEG diversity in comparison to the resting state. In conclusion, the signal diversity of continuous brain signals depends on the stimulated sensory modality, therefore it is not a generic index of the variability of conscious experience.

## Introduction

Which neural mechanisms support state consciousness? In recent work that aimed to address this question, consciousness is typically defined as a dynamic, spatio-temporal process that unfolds in neural networks^1–3^. Accordingly, the most successful approach to predicting global states of consciousness from neural data involves measuring the spatio-temporal diversity of brain activity as a putative correlate of consciousness^4^. A particularly robust and sensitive measure of awareness is the Perturbational Complexity Index (PCI), which measures spatio-temporal integration and the diversity of brain activity by applying the Lempel–Ziv algorithm to the electroencephalographic (EEG) response evoked by a transcranial magnetic stimulation (TMS) pulse^5^. Several studies found that PCI-measured diversity decreases substantially—even at the level of individual participants—during loss of consciousness caused by non-rapid eye movement (NREM) sleep^6,7^ or general anesthesia^8,9^, and in individuals suffering from disorders of consciousness^10,11^. Importantly, PCI did not decrease during rapid eye movement (REM) sleep and ketamine-induced anesthesia—states during which participants are unresponsive but able to experience vivid dreams or hallucinations^5,9^. This finding has been used as an argument that PCI is indeed a specific marker of the subjective experience.

In a related line of work, the Lempel–Ziv algorithm was applied to measure the diversity of continuous brain activity recorded during different states of consciousness. Here Lempel–Ziv provides a measure of signals’ entropy and randomness, rather than spatio-temporal integration and diversity, as in the case of PCI. Nevertheless, the Lempel–Ziv diversity of continuous brain signals has also been shown to successfully predict global states of consciousness. Specifically, the diversity of continuous brain signals decreases during NREM sleep^12,13^, propofol-induced anesthesia^14–16^, and in patients suffering from disorders of consciousness^17^. Further work found that psychoactive substances such as LSD, psilocybin, or ketamine resulted in an *increase* in the diversity of continuous brain activity^18–22^. Importantly, all these substances enhance the intensity and vividness of conscious experiences^23,24^, the magnitude of which was correlated with an increase in signal diversity^18,21,22,25^. Therefore, the observation that EEG diversity not only decreases in states of reduced awareness (in comparison to levels observed during normal wakefulness) but also increases in states related to a more vivid phenomenology has been interpreted as indicating a close relation between diversity measures and the phenomenology of conscious experience.

While measures of brain-signal diversity are robustly related to global states of consciousness, the aforementioned interpretation regarding their specific relation to the subjective experience remains to be appropriately tested^26^. On the phenomenological level, conscious experience is assumed to be both integrated (each experience is a single unified “scene”) and varied, as each moment of conscious experience is unique and different from every other moment^1,27^. Therefore, the diversity of brain activity has been hypothesized to specifically reflect the temporal variability of conscious experience, with higher neural diversity accompanying more-variable experiences^28^. However, when testing this specific hypothesis, conclusions from studies on global states of consciousness are limited by the fact that transitions between conscious and unconscious states (or normal wakefulness and psychedelic states) affect not only consciousness and its correlates but also other neurophysiological and mental processes (i.e., the prerequisites and consequences of consciousness^29,30^). In this respect, studies manipulating the content of consciousness within a given state might be better suited to providing data that supports or falsifies the discussed hypothesis.

Several published studies have already employed such a within-state approach. Boly and colleagues^31^ and Mensen and colleagues^32^ investigated whether brain signal diversity is related to the meaningfulness of perceived visual stimuli (i.e., videos) because, according to their hypothesis, meaningful and temporally structured stimuli cause richer and more temporally diverse conscious experiences than meaningless ones. Both these studies (the former used functional magnetic resonance imaging (fMRI); the latter used EEG) indeed found more diverse cortical responses when participants watched a meaningful video than when they watched one that was scrambled and thus contained less semantic meaning. In line with this, Mensen and colleagues^33^ found that the diversity of EEG-evoked responses was higher when participants saw natural, meaningful images in comparison to artificially generated, meaningless ones. However, in our previous study, in which we used auditory stimuli that varied in terms of information rate and meaningfulness, we found no effect of such manipulation on EEG signal diversity^34^. Relatedly, several studies have compared the diversity of continuous electrophysiological signals recorded during active perception and the resting state: data from three studies indicated that visual perception was related to greater signal diversity than the resting state^21,31,35^, but three studies investigating auditory perception found it was related to *lower* diversity in comparison to the resting state^21,34,35^.

The present study aimed to investigate whether Lempel–Ziv diversity of continuous EEG signals reflects the meaningfulness of perceived stimuli, irrespective of the sensory modality. We reasoned that if signal diversity is a genuine index of the temporal variability of experience, then the relation between diversity and meaningfulness should be the same across modalities (i.e., more meaningful stimuli should always cause greater diversity). Because both modalities have only previously been investigated by separate studies, here we used visual and auditory stimuli in the same experiment and manipulated their meaningfulness in the same way in order to directly compare its effect on EEG diversity. To this end, we recorded EEG signals when participants perceived fragments of videos or audiobooks, both presented in three versions whose meaningfulness varied: original, in which the stimuli were not altered in any way; scrambled, in which the audiobooks and videos were divided into 3-s long fragments and temporally shuffled to reduce semantic information; and noise, in which the stimuli were completely scrambled and thus provided only sensory stimulation without any semantic meaning. Additionally, we recorded resting-state activity to compare signal diversity between rest and active visual and auditory perception. EEG diversity was assessed with two implementations of the Lempel–Ziv algorithm that were introduced and validated in previous studies on global states of consciousness^12,14,18^: one estimating the diversity of the temporal patterns (LZs); the other capturing the diversity of both the spatial and temporal dimensions in one index (LZc).

We hypothesized that the sensory modality would modulate the effect of meaningfulness on Lempel–Ziv EEG diversity. Specifically, we expected that greater EEG diversity would be observed during perception of more meaningful material in the visual modality^31–33^; however, based on our previous work we expected no relation between EEG diversity and meaningfulness in the auditory modality^34^. In line with this, we hypothesized that active visual perception would be related to greater EEG diversity than the resting state^21,31,35^, but active auditory perception would be accompanied by lower diversity than the resting state^21,34,35^.

## Method

### Participants

The sample size was not predefined in the present study. Considering the small sample sizes (between 6 and 9 participants) used in previous studies investigating the relation between the diversity of continuous brain activity and the meaningfulness of visual stimuli^31–33^, we aimed to recruit more than 20 participants for the present study. We thus collected and analyzed data from 24 participants (12 females; age: 22.8 ± 3.2 years). None of the recruited participants were excluded from the analyzed sample. The inclusion criteria were being a native Polish speaker, normal or corrected-to-normal vision and hearing, and no history of neurological, neuropsychiatric, or hearing disorders. This study was designed and conducted in accordance with the Declaration of Helsinki and the STROBE checklist. The experimental procedure was approved by the Committee for Research Ethics at Jagiellonian University in Kraków (KE/07/012018). All participants signed an informed consent document and received monetary compensation of 100 PLN (approximately 20 EUR).

### Auditory and visual stimuli

A commercial audiobook based on a non-fiction Polish novel “Dom na Zanzibarze” (Eng: “A House in Zanzibar”), authored by Dorota Katende and read by a professional female lector (Anna Gajewska) was used to create auditory stimuli for the experiment. When selecting an audiobook, we aimed to choose one that was characterized by contemporary language and was not known to the participants before the experiment. The chosen audiobook met these criteria, as all participants confirmed that they had not read it or heard the selected audiobook before taking part in the experiment.

Episodes of a classic cartoon TV series “Tom & Jerry” from the 1960s were used to create visual stimuli. We aimed to choose video that would seem natural without audio (which excluded the majority of contemporary fiction movies and TV series, in which dialogues correspond with interactions between actors and their mouth movements) and had a simple plot based on a cause–effect relationship. A simple cartoon meets both criteria.

To manipulate the level of meaningfulness of the presented material, we used the same manipulation as used in the previous study by Boly et al.^31^. First, we extracted 30 non-overlapping fragments with a length of 30 s from the audio and video recordings. For each audio and video fragment, we prepared the following versions:

1. *original*: not altered in any way, thus all information was preserved (the most meaningful version);
2. *scrambled*: the original 30-s long fragment was divided into 10 3-s long fragments and their order was randomized, which resulted in partial disturbance of the informativeness and meaning (i.e., the temporal structure was disturbed);
3. *noise*: in case of the visual stimuli, the locations of pixels within each frame of the video (25 frames per second) were randomized, whereas for the auditory stimuli the order of consecutive sounds (frequency of 44,100 Hz) was randomly shuffled. Thus, the noise stimuli preserved the low-level physical features of the original versions but did not represent any semantic information. MATLAB custom scripts were used for all manipulations of the original fragments of the stimuli.

## Experimental procedure

The experimental procedure involved seven conditions, which we refer to as (1) video original; (2) audio original; (3) video scrambled; (4) audio scrambled; (5) video noise; (6) audio noise; (7) resting state. During the experiment, each condition was presented ten times for 30 s (here we also use the term “presentation” when referring to the resting state, even though no stimuli were presented during the resting state). Thus, 300 s of the EEG signal was recorded per condition in total. The order in which the conditions were presented and the assignment of specific audiobook or video fragments to conditions were randomized individually for each participant. Importantly, a given fragment of the audiobook or video was presented only once to each participant (i.e., was used in one version/condition only).

The procedure was written in Presentation^®^ software (Version 20.1, Neurobehavioral Systems, Inc., Berkeley, CA, https://www.neurobs.com) and the experiment was conducted in a sound-attenuated room. A FlexScan EV-2450 (Eizo, Japan) monitor and Sennheiser HD 65 TV (Sennheiser, Germany) headphones were used for stimuli presentation. During each auditory presentation, a white fixation cross was displayed in the center of the screen on a uniform gray background. Participants were asked to fix their gaze on the cross and listen attentively to the recordings through the headphones. The volume was the same for all participants. Video clips were presented in the center of the screen (size: 720 × 576 pixels; subtending 18.4° × 15.0° of the visual angle) on a gray background, and participants were asked to watch the material carefully. During the resting-state condition, participants were asked to just sit still and fixate their gaze on the centrally presented fixation cross, displayed on a gray background. Short breaks were allowed between presentations, and participants were asked to resume the experiment by pressing the space bar whenever they felt ready. During breaks, the same gray background was presented on the screen and there was no audio or video stimulation. The total duration of the presented materials and the resting-state condition was 35 min, while the total duration of the experimental procedure (including breaks) was about 40 min.

### EEG recording and preprocessing

Electroencephalography was recorded from 64 channels (62 scalp sites and reference signal from both earlobes) using a 128-channel amplifier (Quick Amp, Brain Products) and the Brain Vision Recorder software. Gel-filled electrodes made of Ag–AgCl, located on an elastic cap (ActiCap) according to the extended 10–20 system, were used to record EEG signals. The signal was digitized at a sampling rate of 500 Hz. Custom-made Matlab scripts based on the EEGlab functions^36^ were used for preprocessing. Signals were filtered with a 1 Hz high-pass FIR filter (1650 filter order), a 45–55 Hz notch FIR filter (826 filter order), and a 100 Hz low-pass FIR filter (66 filter order). Data was then down-sampled to 250 Hz and divided into 10 s long epochs (210 epochs per participant; 30 per condition). Only data recorded during auditory/visual stimulation or resting-state periods were analyzed; data recorded during breaks were discarded. Based on the visual inspection of EEG signals, the 19.0 ± 15.7 (M ± SD) epochs and 4.2 ± 2,1 channels per participant were discarded due to non-stereotypical artifacts. The numbers of epochs that were removed from the data did not differ statistically between sensory modalities (auditory: 2.96 ± 2.81; visual: 2.38 ± 2.77) or between levels of meaningfulness (original: 2.29 ± 2.63; scrambled: 2.60 ± 2.72; noise: 3.10 ± 3.01). Next, the signals were re-referenced to the average of all channels and decomposed into a number of independent components (equal to the number of retained channels) using Independent Component Analysis, as implemented in the EEGLAB pop_runica function. The Multiple Artifact Rejection Algorithm (MARA^37^) was next used to objectively and automatically identify artifactual components and subtract them from the data (38.9 ± 6.7 components removed per subject). Next, missing channels were interpolated and the signal was re-referenced to an average of the mastoid electrodes’ signals (A1 and A2).

In order to validate that the observed effects were independent of the preprocessing strategy, we conducted a control analysis in which selection of artifactual ICs was performed by the ICLabel^38^ and FASTER^39^ EEGlab plugins. The results of these control analyses were highly consistent with the results of the main analysis, in which the MARA algorithm was used. Details of the control analyses can be found in the Supplementary Material.

### Lempel–Ziv analysis

EEG signal diversity was assessed with two versions of the Lempel Ziv algorithm^40^. To capture the temporal diversity of EEG signals, we used single-channel Lempel–Ziv diversity (LZs). Multi-channel Lempel–Ziv diversity (LZc) was used to capture signal diversity over both space and time simultaneously. We used the algorithmic implementation described in Schartner and colleagues’ studies^14,18^.

To compute LZs diversity, the signal from each EEG channel was assessed independently. First, 10 s long segments of the signal were demeaned and divided by the standard deviation. Next, the linear trend was removed and the envelope of the signal was estimated by applying the Hilbert Transform. The signal was then binarized using the mean value of the envelope as a binarization threshold. Binarized signals were analyzed using the Lempel–Ziv compression algorithm. The last step of computing LZs was normalization of the raw LZs scores. For this purpose, the value from the previous step was divided by the LZs value obtained from the same binary signal but shuffled in time. The obtained LZs values were in the range between 0 (minimal diversity) and 1 (maximal diversity). For the statistical analysis, LZs was averaged over all 62 EEG channels.

To estimate LZc, the 10 s signal segments from each EEG channel were also demeaned and divided by the standard deviation. The linear trend was subtracted from the data and signals were binarized in the same way as for LZs. Then, 62 data series (one for each EEG electrode) were merged into one binary string in the following way: the first 62 digits of the final string are those recorded from all channels at the first time step; the next 62 digits are those recorded at the second time step, etc. The diversity of the obtained binary strings is then assessed in the same way as for LZs.

### Statistical analysis

To test how EEG diversity is related to the meaningfulness of stimuli and the sensory modality in which they are presented, we conducted two repeated-measures ANOVA models: one with LZs, and one with LZc as a dependent variable. In both models, two within-subject factors were included: meaningfulness of a stimulus (3 levels: original, scrambled, noise) and sensory modality (2 levels: visual, auditory). The Greenhouse–Geisser correction was applied when Mauchly’s test indicated a data sphericity violation. To test our hypotheses regarding the differences between active perception and resting state, we compared the measures of diversity from each video and audio condition to the resting state using the paired-samples t-test. Because 6 comparisons were conducted for each diversity measure, the Holm–Bonferroni correction for multiple comparisons was applied. The repeated-measures ANOVA and the paired t-test analyses were also carried out in the Bayesian version to assess the strength of evidence supporting either the null or the alternative hypothesis. JASP software (version 0.16.4)^41^ was used for all statistical analyses.

## Results

### Temporal diversity (LZs)

**Figure 1:**
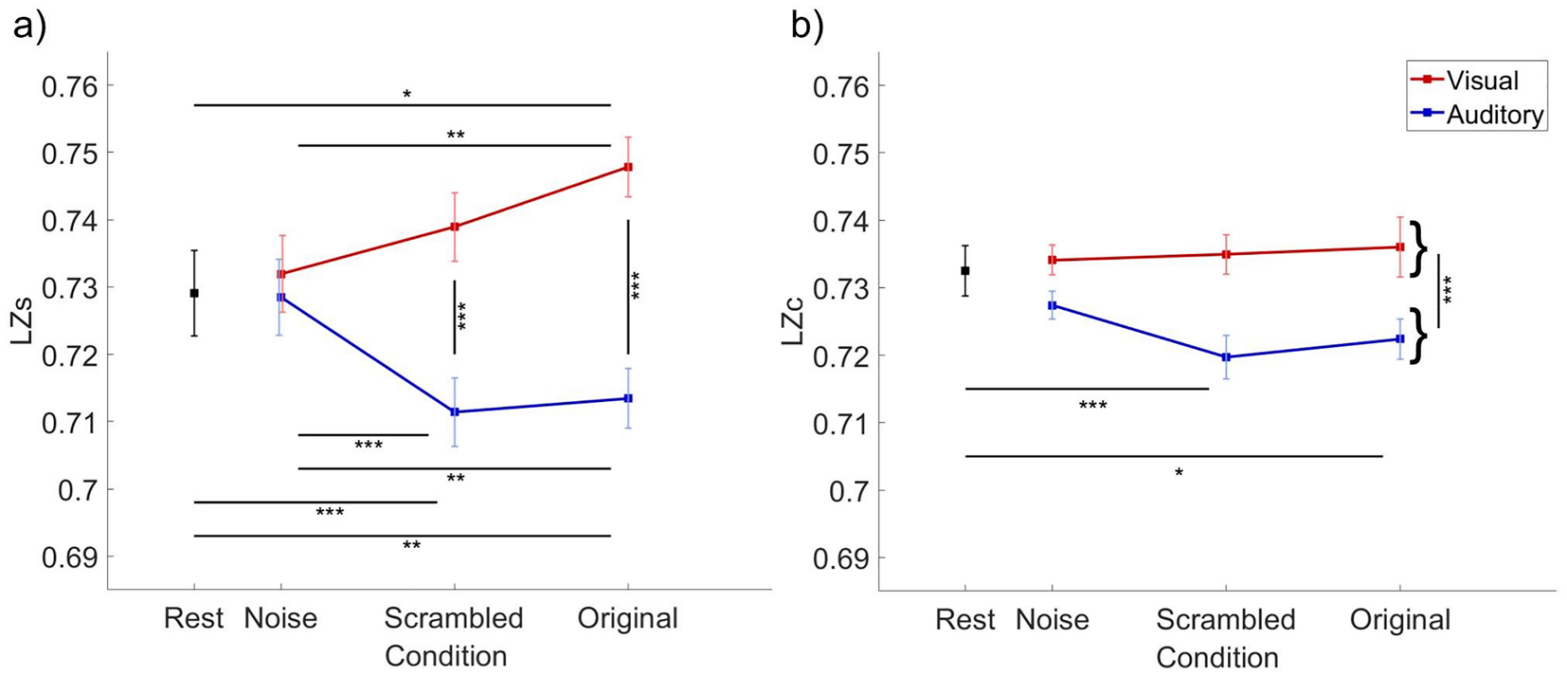
Mean values of (**a**) temporal (LZs) and (**b**) spatio-temporal (LZc) diversity scores observed during the resting state and visual or auditory perception of stimuli varying in meaningfulness (original, scrambled, and noise versions). Error bars represent 95% of the confidence interval. Between-subject variability was removed from error bars^42^. Statistical significance of ANOVA post-hoc comparisons: ∗p < 0.05; ∗∗p < 0.01; ∗∗∗p < 0.001.

When analyzing the temporal diversity of the EEG signal (LZs), we found a significant main effect of the sensory modality (F(1) = 17.674, p < 0.001, η_p_^2^ = 0.435, BF_10_ > 1000; Fig. 1), indicating higher diversity in the visual condition, but no main effect of stimulus meaningfulness (F(1,3) = 2.041; p = 0.161, η_p_^2^ = 0.081, BF_10_ = 0.135). Further, we found an interaction between meaningfulness and sensory modality (F(1,3) = 16.751, p < 0.001, η_p_^2^ = 0.421, BF_10_ > 100), which was explored by conducting the following post-hoc comparisons.

First, we compared different levels of meaningfulness within each modality. Within the visual modality we found higher LZs during perception of the most meaningful original material than during perception of noise (t(23) = 3.864, p = 0.002, *d* = 0.280), but no differences were observed when comparing original with scrambled (t(23) = 2.161, p = 0.167, *d* = 0.157), and scrambled with noise (t(23) = 1.703, p = 0.368, *d* = 0.124). In sharp contrast, in the auditory modality, perception of the original (t(23) = −3.661 , p = 0.004, *d* = −0.266) and scrambled (t(23) = −4.152, p < 0.001, *d* = −0.301) versions resulted in *lower* LZs than perception of noise. Again no difference was observed between the original and scrambled conditions (t(23) = 0.481 , p = 1, *d* = 0.036).

Second, we compared the corresponding levels of meaningfulness across modalities. In comparison to auditory perception, visual perception was associated with higher LZs values in the original (t(23) = 5.626, p < 0.001, *d* = 0.606) and scrambled (t(23) = −4.503 , p < 0.001, *d* = 0.485) conditions, but there was no difference between sensory modalities when the noise material was perceived (t(23) = 0.563, p = 1, *d* = 0.061). The topographic distribution of differences in LZs within and between modalities is presented in Supplementary Fig. 3.

Additionally, we compared the LZs diversity scores between the active perception state and the resting state. In the case of the visual modality, perception of the original material resulted in higher LZs scores than those observed in the resting state (t(23) = 3.390 , p = 0.012, *d* = 0.692, BF_10_ = 15.708), but no differences were found between the scrambled (t(23) = 1.184 , p = 0.216, *d* = 0.384 , BF_10_ = 0.976) and noise (t(23) = 0.909 , p = 0.746, *d* = 0.185, BF_10_ = 0.311) conditions and the resting state. Regarding the auditory modality, *lower* LZs scores were related to perception of the original (t(23) = −4.097, p = 0.002, *d* = −0.836 , BF_10_ = 71.996) and scrambled (t(23) = −5.029, p < 0.001, *d* = −1.027, BF_10_ = 573) materials in comparison to the resting state, but the noise material did not differ from the rest condition (t(23) = −0.170, p = 0.866, *d* = −0.035, BF_10_ = 0.218).

### Spatio-temporal diversity (LZc)

When analyzing the spatio-temporal diversity (LZc) scores, we found a significant effect of the sensory modality (F(1) = 13.401, p < 0.001, η_p_^2^ = 0.368, BF_10_ > 1000; Fig. 1), indicating higher diversity during visual than during auditory perception. However, neither the main effect of meaningfulness (F(2) = 2.144, p = 0.129, η_p_^2^ = 0.085, BF_10_ = 0.131) nor the interaction between meaningfulness and the sensory modality was significant (F(1,466) = 2.713, p = 0.095, η_p_^2^ = 0.106, BF_10_ = 0.48). Therefore, post-hoc comparisons were not conducted.

When comparing active perception to the resting state, we found that auditory perception of original (t(23) = −3.300 , p = 0.015, *d* = −0.674, BF_10_ = 13.019) and scrambled (t(23) = −6.366, p < 0.001, *d* = −1.299, BF_10_ > 1000) stimuli was associated with lower LZc values than during the resting state, but there was no difference between perception of auditory noise and resting state (t(23) = −2.657 , p = 0.056, *d* = −0.542, BF_10_ = 3.636). None of the visual conditions differed from the resting state (original vs resting state: t(23) = 1.177 , p = 0.762, *d* = 0.240, BF_10_ = 0.398; scrambled vs resting state: t(23) = 0.751, p = 0.92, *d* = 0.153, BF_10_ = 0.277; noise vs resting state: t(23) = 0.657, p = 0.92, *d* = 0.134, BF_10_ = 0.261).

## Discussion

The diversity of continuous brain activity constitutes a sensitive and robust neuronal marker of the global states of consciousness^12–18^. Accordingly, it has been proposed that these diversity measures are closely related with conscious experience, specifically with its richness, vividness, and temporal variability^23,24,43,44^. The data collected thus far support this prediction by showing that states of reduced awareness are associated with low signal diversity, normal wakefulness is associated with intermediate diversity levels, whereas psychedelics-induced states are associated with high diversity^4^. However, what limits the conclusions of these studies is that the global states of consciousness differ not only in terms of consciousness per se but also in terms of many accompanying physiological mechanisms that are not directly related to subjective experience. Therefore, we argue that studies using *within-state* manipulations in which these confounds are not as pronounced might provide important evidence that supports or falsifies signal diversity as a correlate of subjective experience.

One of the within-state approaches employed to test whether diversity measures reflect the temporal variability of experience involves manipulating the meaningfulness of stimuli presented to participants. Three studies found that perception of more-meaningful visual stimuli, which is assumed to cause varied experiences, was indeed associated with higher signal diversity than perception of meaningless stimuli, which did not carry much information and thus did not cause varied experiences^31–33^. Our aim was to replicate this finding and extend it by investigating whether the sensory modality in which stimuli are presented modulates the relation between meaningfulness and EEG Lempel–Ziv diversity. Our assumption was that if the diversity of continuous EEG activity is a genuine index of the temporal variability of experience, then its relation to meaningfulness should be the same across modalities. Therefore, we used video fragments as visual stimuli and audiobooks as auditory stimuli, and we manipulated them in the same way to create three versions that varied in meaningfulness, similarly to previous studies^31^. The diversity of the recorded EEG signals when participants were presented with the stimuli was quantified with the Lempel–Ziv algorithm. We report three closely related findings.

### The main findings in the context of previous research on signal diversity

First, we found that the relation between the temporal diversity of continuous EEG activity and the meaningfulness of the perceived stimuli depends on the sensory modality (as indicated by the significant *meaningfulness* × *modality* interaction found for the LZs measure). Specifically, signal diversity increases with increasing meaningfulness in the visual modality, but it *decreases* in the auditory modality. Importantly, the semantic contents of the three versions of stimuli used in our experiment (original, scrambled, noise) differed but were matched in terms of low-level physical features, which cannot thus account for the observed differences between conditions. The effect we found in the visual modality is well in line with the results of previous studies^31–33^, but considering the small sample sizes tested in those studies (between 6 and 9 participants), such replication was of particular importance^45^. However, revealing a reverse effect in the auditory modality, in which exactly the same manipulation was applied, is a novel finding. Importantly, the negative relation between meaningfulness and measures of EEG diversity that we observed challenges the idea that these measures are a genuine correlate of the temporal variability of subjective experience. More generally, our finding points to the limitations of assuming that effects observed in the visual modality will generalize to other sensory modalities^46^.

Secondly and relatedly, we found that perception of meaningful visual stimuli was associated with higher LZs diversity scores than during the resting state, but perception of meaningful auditory stimuli was associated with lower LZs (and LZc) when compared with the resting state. Here, our findings are again well in line with previous studies using resting-state recordings as a baseline, as those studies similarly found greater signal diversity during visual perception^21,31,35^ but *lower* signal diversity during auditory perception^21,34,35^. Importantly, the visual and auditory noise conditions in our study did not differ from the resting state in terms of EEG diversity; this indicates it is the meaning of a stimulus that is crucial for such differences to occur, rather than merely physical stimulation within a given sensory modality.

Finally, we found that visual perception was associated with generally greater EEG diversity than auditory perception, as indicated by the LZs and LZc measures. Previously, only Mediano and colleagues^21^ had compared the diversity of continuous brain activity during perception of visual and auditory stimuli. They observed higher MEG signal diversity when participants were watching a movie in comparison to listening to music with eyes closed, which is generally in line with our findings. However, we argue that the design of our study allows more robust conclusions as participants had their eyes open in both conditions (thus the differences between eyes open and closed cannot account for the observed difference), and the stimuli in both modalities were better matched (i.e., both visual and auditory stimuli had a narrative structure).

Research on brain signal diversity as a correlate of consciousness has largely been inspired by the early work on PCI, which simultaneously measures the balance between diversity and integration in TMS-evoked activity^5^ and is therefore able to directly test the assumptions of the integrated information theory (IIT)^27^. However, studies that have investigated the impact of stimulus meaningfulness applied the Lempel–Ziv algorithm to *continuous* brain activity. It is thus important to emphasize that such application of Lempel–Ziv or other diversity measures captures the randomness and entropy of signals, but it does not reflect the diversity-integration balance as assessed by PCI. The results of these studies should thus be interpreted instead within the recently proposed “weak IIT” framework^47^. Investigating the extent to which PCI reflects the content of consciousness within states will be an important goal for future studies.

### Mechanisms behind modality-dependent effects

How can the differences between sensory modalities observed in our and in previous studies be explained? One interpretation is that vision is the dominant sense through which humans acquire the majority of information about the external world^48,49^ (but see:^46^). Accordingly, a bigger part of the cerebral cortex is devoted to processing visual input than to any other sense^50^. Under this interpretation, a continuous visual stimulus, like the video used here, provides more information and possibly has a stronger effect on the general quality and richness of the integrated subjective experience than a similar auditory stimulus. Therefore, perception of visual stimuli might be associated with generally higher diversity than perception of auditory stimuli (and the resting state), which is what we found. A related aspect (which could, however, be seen as a limitation of our study) is that the visual stimuli that we used contained spatio-temporal information, whereas listening to an audiobook, which is a single stream of speech, mainly requires temporal integration. Future studies might use auditory stimuli of greater complexity, including several actors and background sounds which are better matched to the visual stimuli.

Regarding the decrease of EEG diversity accompanying the increase of meaningfulness in the auditory modality, a phenomenon which can account for this effect is tracking of the speech stream by neural oscillation^51–53^. Under this interpretation, less diverse neural activity will be a consequence of greater synchronization of neural oscillations across the cortex when processing auditory stimuli that are more meaningful and engaging (possibly accompanied by an increase in amplitude of low-frequency oscillations). If this is the case, this shows that modality-specific neural mechanisms play a more important role in defining the quality of conscious experience than the global level of brain-signal diversity.

### Methodological aspects and limitations

The main limitation of this work and previous studies that manipulated the meaningfulness of stimuli is that the effectiveness of such manipulations in affecting temporal variability (or other dimensions) of conscious experience was not measured and directly established^31–33^. In the present study, we did not include any subjective measures as we are not aware of tools which might allow the estimation of concepts such as temporal variability of experience caused by continuous naturalistic stimuli. So far, only studies investigating dream experiences^13,54^ and psychedelics-induced states^18,21,22,25^ have used subjective questionnaires to measure various dimensions of subjective experience (e.g., ego-dissolution, arousal, imagery) and reported that some of them were related to signal diversity^13,18,21,22,25^. Furthermore, several tools have been proposed to measure the contents of consciousness during the resting state, for instance the Amsterdam Resting State Questionnaire^55,56^, and future studies might relate estimates of such questionnaires to signal diversity.

While in studies on global states of consciousness it is impossible to disentangle whether observed differences in neural measures are related to consciousness or some other co-occurring processes (i.e., prerequisites and consequences), within-state manipulations are also affected by the same problem, albeit to a lesser extent. For instance, one might argue that participants in our study engaged more attentional and cognitive resources when meaningful material was presented (i.e., in the original condition) and that the effects on EEG diversity we observed are mainly driven by such a cognitive effect. However, the fact that we observed an interactive effect in a given modality (i.e., a positive relation between diversity and meaningfulness in the visual modality, and negative relation in the auditory modality) indicates that our results cannot be simply explained by greater involvement of attention or any other cognitive process in the meaningful condition.

Different measures have been used to estimate brain signal diversity, and there is no consensus as to which measure is most reliable or sensitive in predicting the global states or the phenomenal aspects of consciousness^57,58^. In the present study, we focused on Lempel–Ziv diversity because it was used in previous works on the relation between meaningfulness and brain signal diversity^31^. We specifically used two implementations of Schartner and colleagues’ Lempel–Ziv algorithm (LZs and LZc), which respectively measured the temporal or spatio-temporal randomness of the binarized EEG signal envelopes^12,14,18^. In our main analysis, both measures show that visual perception is associated with greater diversity than auditory perception, but only LZs indicates an interaction between sensory modality and meaningfulness, which is in line with previous observations that LZs might be more sensitive than LZc (but LZc also indicates an interactive effect in the control analyses that are presented in the Supplement)^18,34^. However, a limitation of both measures is that they measure the randomness of binarized envelopes; thus, they capture only a fraction of the information present in the EEG signal and, second, the effects observed in the envelopes might primarily reflect fluctuations in low-frequency activity (i.e., delta and theta frequency bands). Future studies will conduct more systematic comparisons of how different diversity measures are related to phenomenology, as has already been done for global states of consciousness^17^.

## Conclusions

In conclusion, our study demonstrates that the relationship between the diversity of EEG activity and meaningfulness of the perceived stimulus depends on the sensory modality in which the stimulus is presented. Therefore, our results challenge the diversity of continuous brain activity as a genuine correlate of the temporal differentiation of subjective experience.

## Supplementary Information

Supplementary Information.

## Data Availability

Raw EEG data, aggregated data, and scripts used for data analysis are available from OSF (https://osf.io/e93db/).
