# Supplementary material for: Sensory modality defines the relation between EEG Lempel–Ziv diversity and meaningfulness of a stimulus
Source: Sci Rep. 2023 Mar 1;13:3453. doi: 10.1038/s41598-023-30639-3 (PMC9977735; doi:10.1038/s41598-023-30639-3)
Supplement: Supplementary file 1 — Supplementary Information. [file 41598_2023_30639_MOESM1_ESM.pdf]

Supplementary Material

Sensory modality defines the relation between EEG Lempel-Ziv diversity and meaningfulness of a stimulus

Paweł Orłowski, Michał Bola*

Laboratory of Brain Imaging, Nencki Institute of Experimental Biology of Polish Academy of Sciences, 3 Pasteur Street, 02-093 Warsaw, Poland

Corresponding author:

Michał Bola, PhD
Email: m.bola@nencki.edu.pl
Laboratory of Brain Imaging
Nencki Institute of Experimental Biology
3 Pasteur Street
02-093 Warsaw, Poland

Supplementary Method and Results

In the main analysis reported in the Results section of the manuscript selection of artefactual ICs was performed by the MARA algorithm (38.9 ± 6.67 ICs removed per subject). Therefore, in order to validate that the observed effects are independent of the preprocessing strategy, we conducted a control analysis in which other methods of labeling ICs were used. Specifically, we created control data-sets, for which all preprocessing steps preceding the ICA decomposition and selection of components were the same as for the data set reported in the main analysis, but regarding selection of components we used the following strategies:

1. data-set which was decomposed using ICA algorithm but none of the ICs were removed
2. data-set in which the ICLabel EEGlab plugin was used for detection of artifactual ICs (Pion-Tonachini et al., 2019). The ICLabel plugin was used to identify all components whose source was classified by the pop_icflag() function as an artifact (eye artifacts, heart artifacts, muscle artifacts, channel noise, line noise, or other artifacts) with a probability of 60% or more (26.8 ± 4.19 ICs removed per subject).
3. data-set in which the FASTER EEGlab plugin used for detection of artifactual ICs (Nolan et al., 2010). The component_properties() function from the FASTER plugin was used to calculate the following parameters: the median slope of the time-course of a given IC, the mean slope of the power spectrum of the given IC's time course, the kurtosis of the spatial information in the given IC, and the Hurst exponent of the given IC. Then the z-scores of those parameters were calculated. ICs characterized by z-score exceeding 1.5 for any parameter were removed (13.6 ± 2.52 ICs removed per subject).

Results presented in Supplementary Tables 1 and 2, and Supplementary Figures 1 and 2 indicate that results obtained in the control data-sets are qualitatively similar to those obtained in the main analysis. Specifically, for LZs in all conducted analyses (including the main analysis) we found the main effect of *Modality* and an interaction between *Modality* and *Meaningfulness*. For LZc in the main analysis we found the main effect of *Modality*, but no interaction, whereas in all control analyses both effects, including the interaction, were significant.

LZs	*df*	F	η_p^2	p-value
**No ICs removed**				
Modality	1	150.97	0.87	< 0.001***
Meaningfulness	1.26[a]	1.49	0.06	0.237
Modality:Meaningfulness	1.45[a]	18.12	0.44	< 0.001***
**FASTER**				
Modality	1	19.19	0.46	< 0.001***
Meaningfulness	1.44[a]	2.12	0.09	0.147
Modality:Meaningfulness	1.20[a]	15.94	0.41	< 0.001***
**ICAlabel**				
Modality	1	24.03	0.51	< 0.001***
Meaningfulness	1.49[a]	2.24	0.09	0.133
Modality:Meaningfulness	1.40[a]	18.29	0.44	< 0.001***

Supplementary Table 1. The results of repeated measures ANOVA analysis (dependent variable - LZs) performed on three control data sets. *** - $p < 0.001$; [a] - df corrected with the Greenhouse-Geisser method.

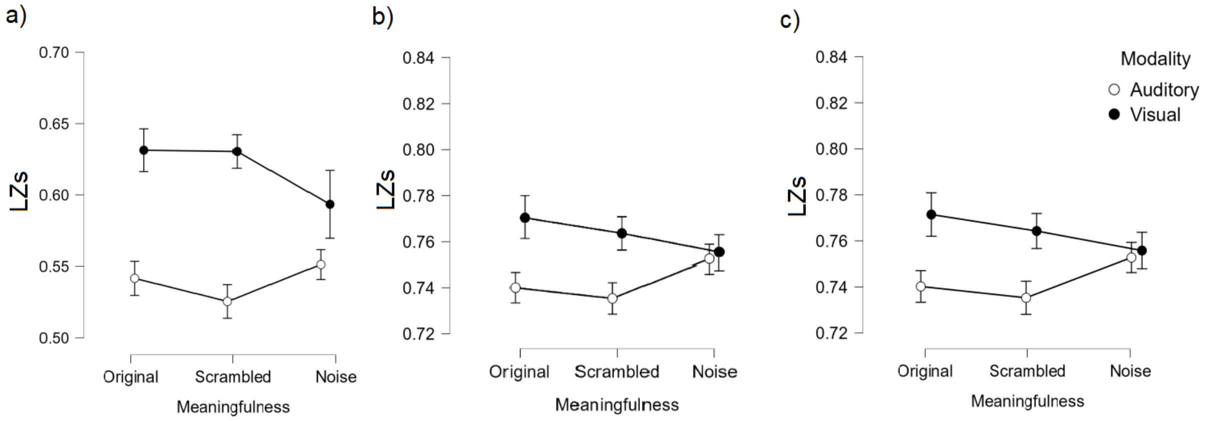

Supplementary Figure 1. Mean values of temporal diversity scores (LZs) observed during perception of visual and auditory stimuli varying in meaningfulness (original, scrambled, and noise versions). a) data from which no ICs were removed; b) FASTER EEGlab plugin used for ICs removal; c) ICLabel EEGlab plugin used for ICs removal. Error bars represent 95% of the confidence interval.

LZc	*df*	F	η_p^2	p-value
**No ICs removed**				
Modality	1	73.80	0.76	< 0.001***
Meaningfulness	1.22[a]	0.76	0.03	0.417
Modality:Meaningfulness	1.44[a]	11.84	0.34	< 0.001***
**FASTER**				
Modality	1	18.53	0.45	< 0.001***
Meaningfulness	1.42[a]	1.58	0.06	0.223
Modality:Meaningfulness	1.39[a]	5.42	0.19	0.017*
**ICAlabel**				
Modality	1	28.18	0.55	< 0.001***
Meaningfulness	1.59[a]	2.64	0.10	0.096
Modality:Meaningfulness	1.44[a]	3.87	0.14	0.043*

Supplementary Table 2. The results of repeated measures ANOVA analysis (dependent variable - LZc) performed on three control data sets. ∗ - p < 0.05; ∗∗∗ - p < 0.001; [a] - df corrected with the Greenhouse-Geisser method.

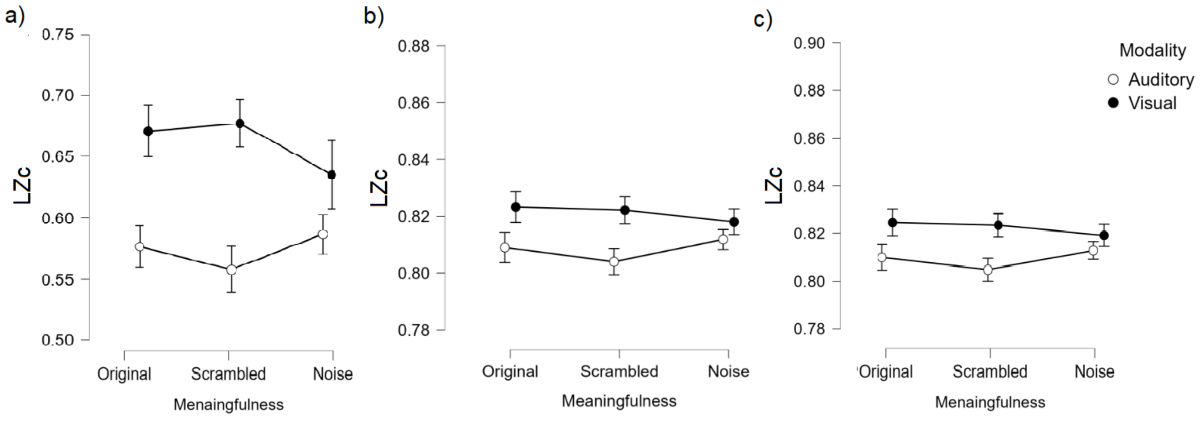

Supplementary Figure 2. Mean values of spatio-temporal diversity scores (LZc) observed during perception of visual and auditory stimuli varying in meaningfulness (original, scrambled, and noise versions). a) data from which no ICs were removed; b) FASTER EEGlab plugin used for ICs removal; c) ICLabel EEGlab plugin used for ICs removal. Error bars represent 95% of the confidence interval.

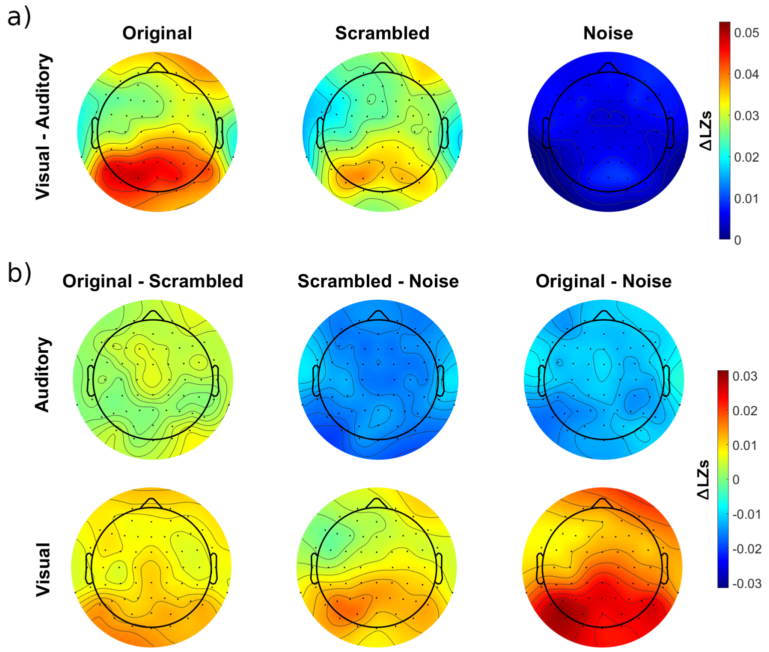

Supplementary Figure 3. Topographic representation of LZs. a) differences in LZs (ΔLZs) between corresponding conditions between modalities b) differences in LZs (ΔLZs) between levels of meaningfulness, within each modality.

References:

1. Pion-Tonachini, L., Kreutz-Delgado, K. & Makeig, S. ICLabel: An automated electroencephalographic independent component classifier, dataset, and website. *NeuroImage*. **198**, 181-197. https://doi.org/10.1016/j.neuroimage.2019.05.026 (2019).

2. Nolan, H., Whelan, R. & Reilly, R. B. FASTER: fully automated statistical thresholding for EEG artifact rejection. *Journal of neuroscience methods*. **192**, 152-162. https://doi.org/10.1016/j.jneumeth.2010.07.015 (2010).
